# Supplementary material for: Response of conventional sunflower cultivars to drift rates of synthetic auxin herbicides
Source: PeerJ. 2024 Jan 10;12:e16729. doi: 10.7717/peerj.16729 (PMC10787541; doi:10.7717/peerj.16729)
Supplement: Supplemental Information 4 [file peerj-12-16729-s004.pdf]

QUALITY PARAMETERS OF IRRIGATION WATER

PARAMETER	RESULT	UNIT
pH:	7.66	-
ELECTRICAL CONDUCTIVITY (EC):	749.97	µS/cm
SODIUM (Na):	19.27	ppm
POTASIUM (K):	15.2	ppm
CALCIUM (Ca):	1,7	me/L
MAGNESIUM (Mg):	1.9	me/L
CARBON TRIOXIDE (CO_3):	1.2	me/L
BICARBONATE (HCO_3):	1.8	me/L
CLOR (Cl):	1.2	me/L
SULPHATE (SO_4):	3.31	me/L
SODIUM ADSORPTION RATE (SAR):	0.63	-
RESIDUAL SODIUM CARBONATE (RSC)	-0.6	me/L
%Na	25.42	-
